# Whole genome sequence data of *Streptomyces californicus* TBG-201, a chitinolytic actinomycete isolated from the Vandanam sacred groves of Alleppey District, Kerala, India

**DOI:** 10.1016/j.dib.2023.109228

**Published:** 2023-05-11

**Authors:** Kumaradasan Sreelatha Deepthi, Sajna Salim, Anandhavally Satheesan Anugraha, Shiburaj Sugathan

**Affiliations:** a Department of Microbiology, Government Arts and Science College Nattukal, Kozhinjampara, Palakkad, Kerala, Pin- 678554, India; b Department of Biotechnology, University of Kerala, Kariavattom Campus, Trivandrum, Kerala, Pin- 695581, India; c Department of Botany, University of Kerala, Kariavattom Campus, Trivandrum, Kerala, Pin- 695581, India

**Keywords:** *Streptomyces californicus*, Draft-genome, Chitinase, Secondary metabolites, CAZyme, AntiSMASH

## Abstract

This study presents the complete genome sequence of *Streptomyces californicus* TBG-201 isolated from the soil samples of Vandanam sacred groves in Alleppey District, Kerala, India. The organism has potent chitinolytic activity. The genome of *S. californicus* TBG-201 was sequenced using the Illumina HiSeq-2500 platform with 2 × 150bp pair-end protocol and assembled using Velvet version 1.2.10.0. The assembled genome has a 7.99 Mb total length, a G+C content of 72.60%, and 6683 protein-coding genes, 116 pseudogenes, 31 rRNAs, and 66 tRNAs. AntiSMASH analysis revealed abundant biosynthetic gene clusters, while the dbCAN meta server was used to detect carbohydrate-active enzyme coding genes. The NCBI Prokaryotic Genome Annotation Pipeline was used for genome annotation. The presence of numerous genes coding for chitin degradation indicates the chitinolytic ability of this strain. The genome data have been deposited in NCBI with the accession number JAJDST000000000.

**Specifications Table**

**Table:** WGS of *Streptomyces californicus* TBG-201: Specifications table

Subject	Biological science
Specific subject area	Microbiology, Bacterial Genomics, Microbial biotechnology.
Type of data	Whole genome sequence data, predicted genes, and functional analysis of respective proteins, figure, table.
How the data were acquired	De novo sequencing was performed using the Illumina HiSeq-2500 sequencing platform. Genome assembled using ABySS v. 2.0.1, MaSuRCA v. 2.3.2, and Velvet v. 1.2.10. Genome annotation was done by the NCBI Prokaryotic Genome Annotation Pipeline (PGAP).
Data format	Raw and analyzed.
Description of data collection	The modified CTAB method was used to extract the genomic DNA of *S. californicus* TBG-201, and a genomic library was prepared using the Illumina TruSeq Nano DNA Library Prep Kit. The genome was sequenced on the Illumina HiSeq-2500 platform, utilizing a 2 × 150bp pair-end protocol. Genome assembly was carried out using ABySS version 2.0.1, MaSuRCA version 2.3.2, and Velvet version 1.2.10. The NCBI Prokaryotic Genome Annotation Pipeline (PGAP) was employed to perform genome annotation, utilizing the best-placed reference protein set. Prediction of biosynthetic gene clusters was conducted using AntiSMASH, while identification of carbohydrate-active enzymes was performed using CAZy analysis via the dbCAN meta server.
Data source location	• Institution: Department of Botany, Kariyavattom Campus, University of Kerala, Trivandrum, Kerala, India. • City/Town/Region: Vandanam sacred groves, Alleppey District, Kerala. • Country: India. • Latitude and longitude for collected samples/data: 9.4946^o^N, 76.3311^o^E. Altitude- 6 m.
Data accessibility	Repository name: NCBI GenBank Bioproject: PRJNA772892 NCBI BioSample: SAMN22418706 NCBI GenBank Accession Number: JAJDST000000000 Assembly: ASM2064085v1 Direct URL to data: https://www.ncbi.nlm.nih.gov/nuccore/JAJDST000000000 All additional data and supplementary files may be accessed at Mendeley data: https://data.mendeley.com/datasets/fgtz42yfh7, DOI:10.17632/fgtz42yfh7.1

## Value of the Data

• The isolate *S. californicus* TBG-201 is a potent chitinase producer, which makes it a significant candidate for biotechnological applications. The genome contains genes coding for chitin degradation. The presence of the GH19 chitinase gene shows that it can produce family-19 chitinases, which are very similar to plant chitinase-C. Chitinase-19 has received much attention recently because of its potential use in the biocontrol of phytopathogens like insects and fungi.
• Thirty-five biosynthetic gene clusters were identified from the genome using AntiSMASH, which suggests the potential of the organism to produce a wide range of secondary metabolites. Various carbohydrate-active enzymes were identified in the genome by CAZy analysis which provides an understanding of the organism's carbohydrate metabolism and potential biotechnological applications. The genome data can be used for elucidating specific genomic and functional analysis.
• Whole genome sequence data of *S. californicus* TBG-201 can benefit researchers and scientists for functional genomics and enzyme research. The data also provide insights for the researchers on the potential applications of *S. californicus* TBG-201.
• The genome sequence data of *S. californicus* TBG-201 can be primarily used for research on various biotechnological applications. The presence of several gene clusters, genes for chitin degradation, and other carbohydrate-active enzymes in the genome indicates the organism's ability to produce numerous secondary metabolites and degrade chitin and other complex carbohydrates which may be experimentally studied.

## 1 Objective

*S. californicus* TBG-201 was isolated in our laboratory from the soil samples of Vandanam sacred groves of Alleppey District in Kerala and was found to be a potent chitinase producer. The organism's whole genome was sequenced to understand better the genetic basis of the isolate's chitinolytic activity. The genome assembly was annotated using NCBI PGAP to identify the protein-coding genes, rRNAs, tRNAs, and pseudogenes. The biosynthetic gene clusters were identified using antiSMASH, which suggested the potential of the organism to produce a broad spectrum of secondary metabolites. The genes for carbohydrate-active enzymes were identified using CAZy analysis. Overall, the generation of this dataset was motivated by the need to understand the genetic basis of the chitinolytic activity of *S. californicus* TBG-201, which has potential biotechnological applications.

## 2 Data Description

Whole genome sequence data of the chitinolytic actinomycete, *S. californicus* TBG-201, is reported here. The pre-processing of data after quality control gave 3,976,878 reads with 555.71MB of base pairs for R1 and 503.25MB of base pairs for R2. The de novo assembly resulted in 50 scaffolds, 129 contigs, and an N50 value of 154,990. Velvet assembly was done using a k-mer value of 79, resulting in a genome with 7,994,281 base pairs with a genome coverage of 99.5x. The BUSCO score was C: 95.3% (S: 93.9%, D: 1.4%, F: 0.7%, M: 4.0%, N: 148). The sequence was deposited in GenBank under the accession number JAJDST000000000. The functional annotations and gene predictions using the NCBI prokaryotic genome annotation pipeline are available at GenBank. The general features of the genome assembly are given in Table 1. The genes coding for proteins associated with chitin degradation in *the S. californicus* TBG-201 genome, as obtained from NCBI PGAP annotation, are shown in Table 2.

**Table 1:** The general characteristics of the *S. californicus* TBG-201 genome.

Features	*S. californicus TBG-201*
Total sequence length (bp)	7,994,281bp
Total un-gapped length (bp)	7,988,965bp
Number of scaffolds	50
Gaps between scaffolds	0
Scaffolds N50	1,079,985
Scaffolds L50	3
Number of contigs	129
Contig N50	154,990
Contig L50	15
G + C content (%)	72.60%
Genes (Total)	6,899
CDSs (Total)	6,799
Genes (coding)	6,683
CDSs (with protein)	6,683
Genes (RNA)	100
rRNAs	7, 10, 14 (5S, 16S, 23S)
Complete rRNAs	5, 5 (5S, 23S)
Partial rRNAs	2, 10, 9 (5S, 16S, 23S)
tRNAs genes	66
ncRNAs	3
Pseudo genes (total)	116
CRISPR Arrays	2
Number of component sequences (WGS)	50

**Table 2:** Genes for chitin degradation identified from *S. californicus* TBG-201 genome.

Enzyme	GenBank Accession	Product Name
Chitinases	MCC0576132.1	GH18, Chitinase D- Exochitinase
	MCC0576640.1	GH18 type II chitinase C- Endochitinase and CBM_2
	MCC0577086.1	GH18 type II chitinase C- Endochitinase and CBM_2
	MCC0578779.1	GH18 type II chitinases ChiA, ChiC and ChiC_BD
	MCC0576765.1	GH18 chitinase D- Exochitinase and CBM_4_9
	MCC0577358.1	GH18 type II chitinases- Endochitinase
	MCC0577439.1	GH18 Chitinase D Exochitinase and CBM_4_9
	MCC0580136.1	GH19, chitinase class I and ChiC_BD
	MCC0580137.1	GH19, chitinase class I and ChiC_BD
Deacetylases	MCC0574417.1	CE4- polysaccharide deacetylase
	MCC0574556.1	CE4- polysaccharide deacetylase
	MCC0575334.1	CE4- polysaccharide deacetylase
	MCC0577008.1	CE4- NodB_like_6s_7s domain-containing- polysaccharide deacetylase
	MCC0577677.1	CE4- polysaccharide deacetylase
	MCC0577144.1	CE4- polysaccharide deacetylase
	MCC0578155.1	N-acetylglucosamine-6-phosphate deacetylase
N-acetyl glucosaminidase (NAGase)	MCC0579983.1	GH20 beta-N-acetyl glucosaminidase domain-containing protein
	MCC0574893.1	GH20- Chitobiases, beta-N-acetyl hexosaminidase
	MCC0577590.1	GH20 glycosyl hydrolase
β Galactosidase	MCC0576278.1	GH 2- beta-galactosidase
	MCC0579319.1	GH3- beta-galactosidase
β Glucosidase	MCC0574707.1	GH 3- Periplasmic beta-glucosidase
	MCC0576914.1	GH3, Periplasmic beta-glucosidase, CBM_11
	MCC0577205.1	GH3, Periplasmic beta-glucosidase
	MCC0578016.1	GH3 Periplasmic beta-glucosidase and CBM6
	MCC0574753.1	beta-glucosidase
	MCC0575370.1	beta-glucosidase
	MCC0580340.1	GH1 beta-glucosidase
Chitosanase	MCC0577157.1	GH5 glycosyl hydrolase
	MCC0575462.1	GH5 protein- endoglucanase/ cellulase
Glucokinase	MCC0575527.1	ROK family glucokinase
	MCC0579445.1	ROK family glucokinase
Glucosamine 6-phosphate deaminase	MCC0577204.1	glucosamine-6-phosphate deaminase
Lytic chitin monooxygenase	MCC0574750.1	lytic polysaccharide monooxygenase
	MCC0574783.1	lytic polysaccharide monooxygenase
	MCC0576047.1	lytic polysaccharide monooxygenase
	MCC0577833.1	lytic polysaccharide monooxygenase
	MCC0579060.1	lytic polysaccharide monooxygenase
Chitinase sensor kinase	MCC0575518.1	two-component sensor histidine kinase
	MCC0577752.1	two-component sensor histidine kinase
	MCC0578211.1	two-component sensor histidine kinase
	MCC0580129.1	two-component sensor histidine kinase
Two-component system response regulator protein	MCC0574951.1	two-component system response regulator MtrA
	MCC0577530.1	two-component system response regulator AfsQ1

The annotation of the constitutive modules of CAZymes from the gene sequence is primarily used to assess and identify an organism's capacity to produce complex carbohydrate-degrading enzymes. The meta server dbCAN combines three cutting-edge tools for CAZome annotation: (i) HMMER search against the dbCAN HMM (hidden Markov model) database; (ii) DIAMOND search against the CAZy pre-annotated CAZyme sequence database; and (iii) Hotpep search against the conserved CAZyme short peptide database. The three methods' outputs were combined to get the best possible results from automated CAZyme annotation. Only the ones detected by at least two methods were selected and given in Table 3.

**Table 3:** CAZy count of *S. californicus* TBG-201.

CAZy Function class	CAZy Family (No.)
Auxiliary activity	AA10 (5), AA3 (1), AA5 (1)
Carbohydrate-binding module	CBM11 (1), CBM12 (3), CBM13 (5), CBM16 (2), CBM2 (2), CBM20 (1), CBM25 (1), CBM32 (9), CBM35 (2), CBM41 (1), CBM42 (1), CBM48 (5), CBM0 (1), CBM6 (1), CBM5 (4), CBM50 (8), CBM51 (1)
Carbohydrate esterase	CE14 (5), CE4 (5), CE9 (1)
Glycoside hydrolases	GH0 (2), GH1 (3), GH101 (1), GH109 (1), GH114 (1), GH135 (1), GH136 (1), GH13 (13), GH15 (2), GH154 (1), GH16 (2), GH171 (1), GH18 (7), GH19 (2), GH2 (1), GH20 (2), GH23 (7), GH25 (2), GH29 (1), GH3 (3), GH31 (1), GH33 (1), GH35 (1), GH4 (2), GH43 (1), GH5 (2), GH6 (2), GH64 (2), GH65 (1), GH77 (1), GH81 (1), GH84 (1), GH87 (2), GH92 (1)
Glycosyl transferases	GT1 (5), GT2 (28), GT20 (1), GT28 (2), GT35 (1), GT39 (1), GT4 (13), GT51 (4), GT81 (1), GT83 (3), GT87 (1)
Polysaccharide lyases	PL31 (1), PL8 (1)

Thirty-five biosynthetic gene clusters, including those for antibiotics, melanin, antifungal compounds, siderophore, geosmin, carotenoid, osmolyte, and terpenes, were identified using the AntiSMASH tool (Table 4). Many of them codes for secondary metabolites that have less than 20% similarity to known compounds. That indicates the novelty of metabolites offering the possibility of discovering new bioactive compounds.

**Table 4:** Secondary metabolite clusters of *S. californicus* TBG-201 as determined by antiSMASH.

Region	Type	The most similar known cluster	Similarity %
Region 3.1	T1PKS, NRPS	Kanamycin	2%
Region 3.2	Phosphonate	Rhizocticin A	9%
Region 4.1	Siderophore	Ficellomycin	3%
Region 5.1	NRPS, T3PKS	Tetronasin	11%
Region 5.2	Melanin	Melanin	100%
Region 5.3	NRPS	Ibomycin	7%
Region 5.4	NRPS, T1PKS	SGR PTMs	100%
Region 6.1	Lanthipeptide-class-ii and iii	-	-
Region 6.2	Siderophore	Desferrioxamin B	100%
Region 6.3	Thiopeptide, LAP	-	-
Region 7.1	NRPS	Kanamycin	1%
Region 7.2	RiPP-like	-	-
Region 7.3	Other, NRPS	Mitomycin	16%
Region 7.4	NRPS-like, ladderane	Atratumycin	39%
Region 7.5	Terpene	Hopene	69%
Region 7.6	NRPS-like, NRPS	Viomycin	100%
Region 8.1	Butyrolactone	Coelimycin P1	12%
Region 8.2	Terpene	Geosmin	100%
Region 8.3	NRPS	Streptobactin	94%
Region 8.4	NRPS	Coelichelin	81%
Region 8.5	T3PKS	Herboxidiene	6%
Region 8.6	NRPS-like	-	-
Region 9.1	Terpene	-	-
Region 9.2	Lanthipeptide-class-iii	AmfS	100%
Region 9.3	T1PKS	-	-
Region 9.4	Melanin	Melanin	100%
Region 9.5	Lanthipeptide-class-i	-	-
Region 10.1	Terpene	Isorenieratene	100%
Region 11.1	Ectoine	Ectoine	100%
Region 11.2	T2PKS	Griseorhodin A	100%
Region 12.1	Butyrolactone, Ectoine	Showdomycin	47%
Region 12.2	Lasso peptide	Keywimysin	100%
Region 12.3	Lanthipeptide-class-i	-	-
Region 12.4	NRPS-like	WS9326	7%
Region 12.5	RRE-containing	-	-

**Fig. 1:**
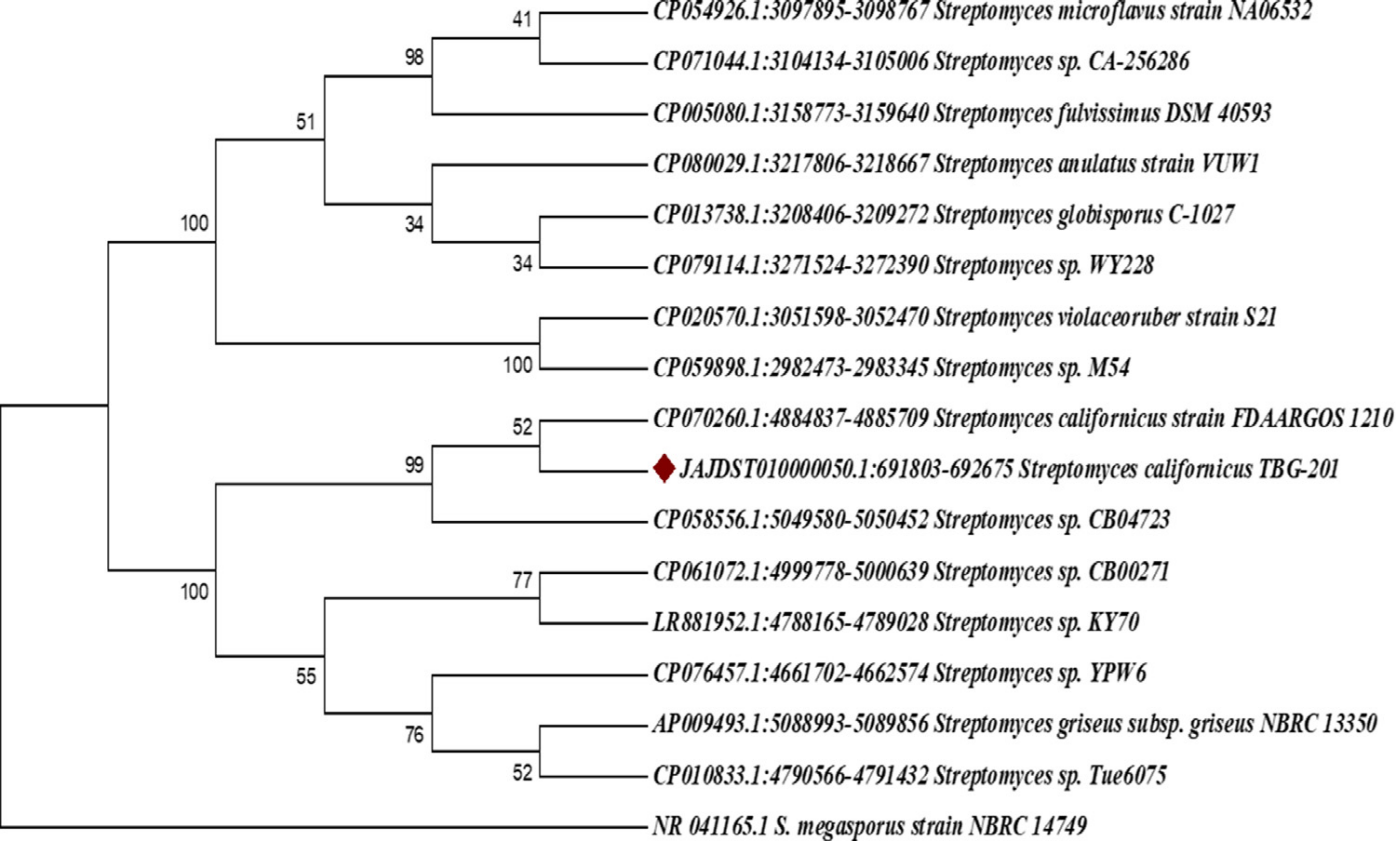
Evolutionary relationships of taxa of *S. californicus* TBG-201 based on 16S rDNA. Using the Neighbor-Joining method, the evolutionary history was deduced [4]. The bootstrap consensus tree inferred from 1000 replicates represents the evolutionary history. Next to each branch is the percentage of replicate trees in which the related taxa grouped together in the bootstrap test (1000 replicates) [5]. The evolutionary distances were calculated using the Jukes-Cantor method [6] and are in the units of the number of base substitutions per site. The analysis involved 17 nucleotide sequences. Codon positions included were 1st+2nd+3rd and noncoding. For each sequence pair, the ambiguous positions were eliminated. There were a total of 1474 positions in the final dataset.

**Fig. 2:**
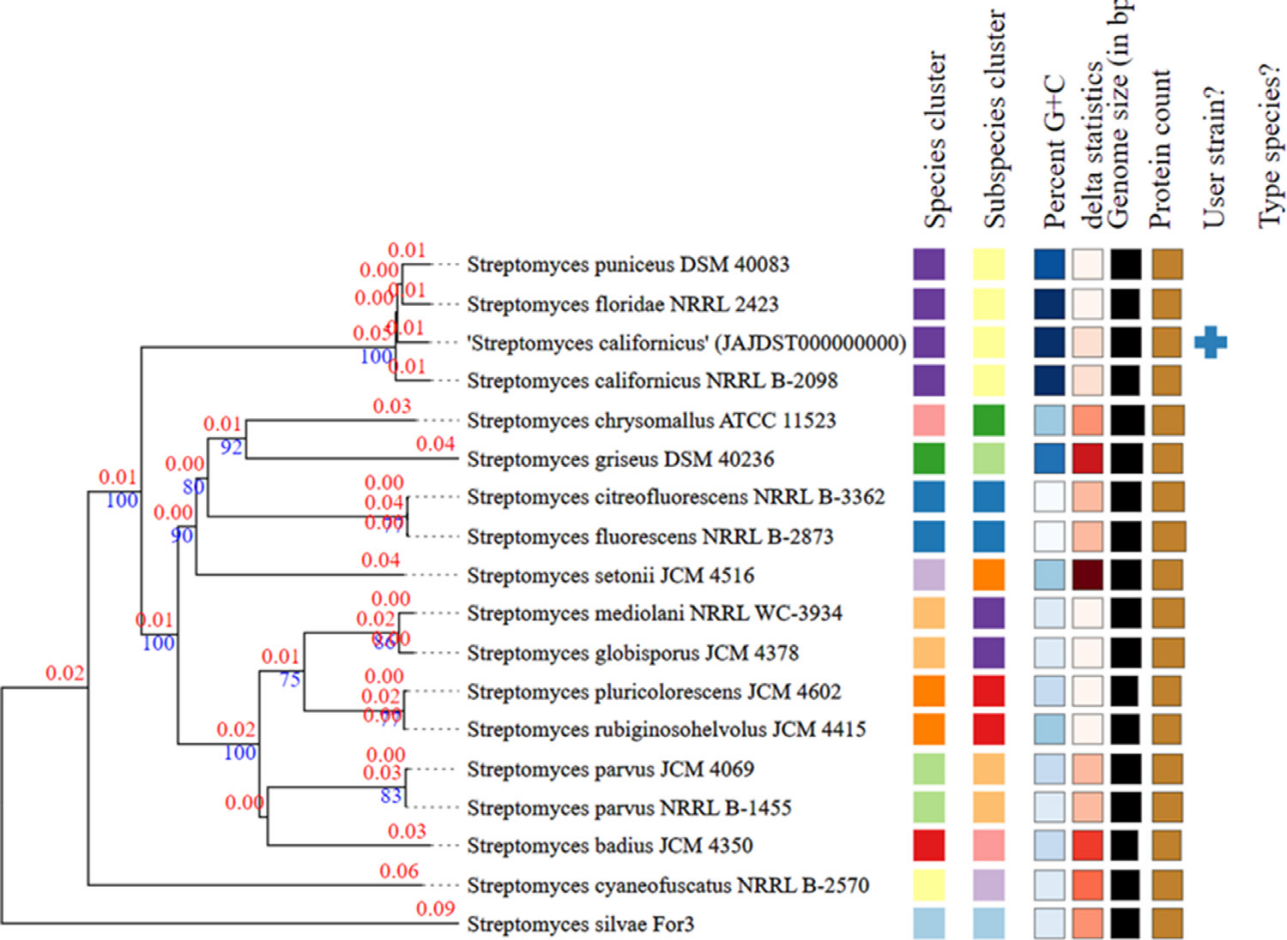
TYGS GBDP phylogeny of *S. californicus* TBG-201 based on genome data. The phylogenetic tree for strain TBG-201 was inferred using FastME 2.1.6.1 [7]. GBDP pseudo-bootstrap support values above 60% from 100 replications with branch support of 78.7% are shown above the branches. The tree has a δ statistics value of 0.096 - 0.221.

**Fig. 3:**
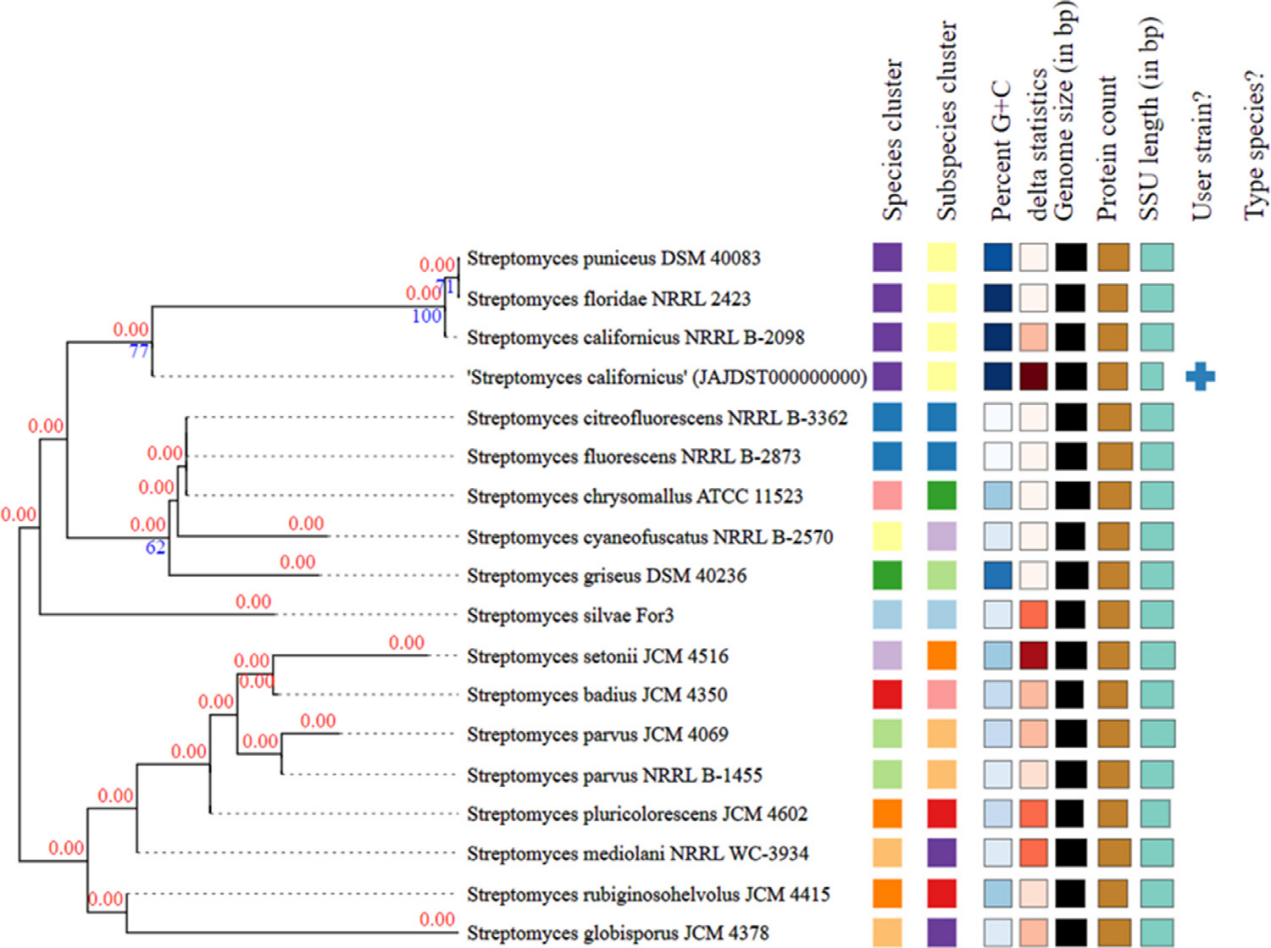
TYGS GBDP Phylogeny of *S. californicus* TBG-201 based on 16s data. The phylogenetic tree was constructed with FastME 2.1.6.1 [7] using GBDP distances assessed from 16S rDNA gene sequences. The digits above branches represent the GBDP pseudo-bootstrap support value > 60% from 100 replications, with average branch support of 46,1%. δ statistics value of the tree was found to be 0.264 - 0.446.

The neighbor-joining tree based on 16S rDNA gene sequences shows that the strain TBG-201 is highly similar to *S. californicus* strain FDAARGOS 1210 (Fig. 1). To confirm the taxonomic identity of strain TBG-201, digital DNA-DNA hybridization (dDDH) was done. The dDDH values d4 for *S. puniceus* strain DSM 40083 and *S. floridae* NRRL 2423 are 88.4% for both. *S. puniceus* [1] and *S. floridae* [2] are synonyms for *S. californicus* [3]. The strain TBG-201 (JAJDST000000000) belongs to the known species *S. californicus* (Fig. 2, Fig. 3)*.* The average nucleotide identity (ANI) value of *S. californicus* TBG-201 was found to be 98.65% with *S. californicus* strain FDAARGOS_1210 and 97.69% with *Streptomyces* sp. CB04723, the closest phylogenetic neighbors. These values are higher than the generally accepted species threshold level of 96%, indicating that the strain TBG-201 (JAJDST000000000) belongs to the known species *S. californicus*.

## 3 Experimental Design, Materials and Methods

### 3.1 Culture maintenance

*S. californicus* TBG-201 was grown and maintained on ISP2 agar media (Yeast extract Malt extract agar) at 28 ± 2°C. Stock cultures were maintained at -80°C in a 50% glycerol stock.

### 3.2 Genomic DNA extraction

*S. californicus* TBG-201, grown in YEME Medium with 34% sucrose and 0.5% glycine, was used to isolate high molecular weight genomic DNA for whole genome sequencing. The organism was incubated at 28 ± 2°C at 180 rpm for five days, and the genomic DNA was extracted using the CTAB method [8].

### 3.3 Genome sequencing, data pre-processing, and *De Novo* assembly

Library preparation was done using the Illumina TruSeq Nano DNA Library Prep Kit (Nextera mate-pair library prep kit). The Illumina HiSeq 2500 sequencing platform with a 2 × 150bp pair-end protocol was used for doing the De novo sequencing of the genome. A fastq quality check was carried out for average base content per read, base quality score distribution, and G+C distribution in the reads. The fastq files were pre-processed using AdapterRemovalV2 v2.3.1 (https://github.com/mikkelschubert/adapterremoval) and filtering out the reads with an average quality score of less than 30 from the paired-end reads using Cutadapt v1.8 [9]. FastUniq v1.1 (https://sourceforge.net/projects/fastuniq/files/ ) was used to remove the duplicate reads [10]. De novo Assembly was done using AbySS v2.0.1 (https://github.com/bcgsc/abyss), MaSuRCA v2.3.2 (http://www.genome.umd.edu/masurca.html), SPades, and Velvet v1.2.10 (http://www.mybiosoftware.com/velvet-1-1-07-sequence-assembler-short-reads.html) [11]. BUSCO v2 (http://busco.ezlab.org/) was used to check if assembled contigs have conserved genes [12].

### 3.4 Sequence submission to NCBI, annotation, and analysis

The genome sequence was submitted to the NCBI through its genome submission portal (https://submit.ncbi.nlm.nih.gov/subs/genome/). The genome annotation was done by NCBI Prokaryotic Genome Annotation Pipeline (PGAP) using the best-placed reference protein set, the GeneMarkS-2+ annotation method [13]. The annotated genes were searched manually to identify the genes involved in chitin degradation. Carbohydrate-active enzymes (CAZyme) annotation was performed using the dbCAN meta server (https://bcb.unl.edu/dbCAN2/blast.php) [14]. The presence of biosynthetic gene clusters (BGCs) in the genome was predicted using the AntiSMASH 6.0.1 server (https://antismash.secondarymetabolites.org/#!/start) [15].

### 3.5 Phylogenetic and comparative genomic analysis

The gene sequence encoding the 16S rDNA of *S. californicus* TBG-201 was retrieved from GenBank. The NCBI BLAST tool (https://blast.ncbi.nlm.nih.gov/Blast.cgi) was used to retrieve closely related sequences from GenBank, and similar sequences were then aligned using the ClustalW. MEGA6 was used to construct the evolutionary tree [16]. Type Strain Genome Server (TYGS) (http://tygs.dsmz.de) was used for whole genome-based taxonomy analysis [17]. The average Nucleotide Identity (ANI) value was calculated using CJ Bioscience's online Average Nucleotide Identity calculator that uses the OrthoANIu algorithm (https://www.ezbiocloud.net/tools/ani) [18].

## Ethics Statements

Not applicable.

## CRediT authorship contribution statement

**Kumaradasan Sreelatha Deepthi:** Methodology, Formal analysis, Investigation, Writing – original draft. **Sajna Salim:** Resources, Validation. **Anandhavally Satheesan Anugraha:** Writing – review & editing. **Shiburaj Sugathan:** Conceptualization, Funding acquisition, Project administration, Supervision.

## Declaration of Competing Interest

The authors of this paper state that they do not have any financial or personal interest that could have influenced their work or created a conflict of interest.

## Data Availability

Whole genome sequence data of a chitinolytic actinomycete, Streptomyces californicus TBG-201 (Original data) (Mendeley Data).
